# Evaluation of the Effects of Anti‐Aβ Antibody Drug Treatment on Brain Atrophy Using a Voxel‐Based Specific Regional Analysis System for Alzheimer's Disease

**DOI:** 10.1002/alz70856_098835

**Published:** 2025-12-24

**Authors:** Yuta Inagawa, Yuki Miyagi, Shoya Inagawa, Naoto Takenoshita, Tomohiko Sato, Mana Yoshimura, Kazuhiro Saito, Haruo Hanyu, Soichiro Shimizu

**Affiliations:** ^1^ Tokyo Medical University, Shinjuku‐ku, Tokyo, Japan

## Abstract

**Background:**

The voxel‐based specific regional analysis system for Alzheimer's disease (VSRAD) is a cutting‐edge imaging tool used to assess and diagnose Alzheimer's disease (AD), by analyzing structural changes in the brain using MRI. In this study, we aimed to evaluate the effects of lecanemab on hippocampal and overall brain atrophy, by examining changes using VSRAD.

**Method:**

This study included 16 patients. All patients were diagnosed as having probable AD based on the National Institute on Aging and Alzheimer's Association diagnostic criteria in 2011. Aβ pathology was confirmed either by amyloid positron emission tomography or cerebrospinal fluid analysis, and lecanemab treatment was initiated. For VSRAD analysis, three dimensions sagittal T1‐weighted spin‐echo images were obtained. VSRAD Advance 2 software was used to assess the severity of atrophy. The volume of interest (VOI) to be used as a reference in the assessment of brain atrophy in patients with AD was the medial temporal cortex (hippocampus, amygdala, and most of the olfactory cortex). The severity of VOI atrophy was assessed by comparing scores obtained before the first lecanemab administration, and scores obtained before the fifth administration. Additionally, the proportion of atrophy within the VOI relative to whole brain atrophy was evaluated.

**Result:**

Mean severity of VOI atrophy before the first administration was 1.61 ± 0.76, whereas that before the fifth administration was 1.38 ± 0.66, demonstrating a statistically significant difference (*p* < 0.001). In terms of the VOI/whole brain atrophy ratio, the mean ratio before the first administration was 6.76 ± 6.44, whereas that before the fifth administration was 4.92 ± 4.83, also showing a statistically significant difference (*p* < 0.05).

**Conclusion:**

Our results demonstrated that the severity of VOI atrophy measured using VSRAD was significantly decreased in patients treated with lecanemab in actual clinical practice.

